# Draft Genome Sequence of Oenococcus oeni Strain X_2_L (CRL1947), Isolated from Red Wine of Northwest Argentina

**DOI:** 10.1128/genomeA.01376-14

**Published:** 2015-01-02

**Authors:** Lucía M. Mendoza, Lucila Saavedra, Raúl R. Raya

**Affiliations:** Centro de Referencia para Lactobacilos (CERELA), Tucumán, Argentina

## Abstract

We report the draft genome sequence of Oenococcus oeni strain X_2_L, a potential starter culture of malolactic fermentation, isolated from Malbec wine of Argentina. Genes encoding for enzymes involved in the metabolism of malate, citrate, and nitrogen compounds, as well as aroma compounds, were found in this genome, showing its ability to improve the sensorial characteristics of wines.

## GENOME ANNOUNCEMENT

Oenococcus oeni is the best-adapted wine lactic acid bacterium and is almost exclusively used for the induction of malolactic fermentation (MLF), the conversion of malic acid into lactic acid during the wine-making process (1). MLF is a critical step to improve the quality of wine because acidity decreases, microbiological stability increases, and sensory characteristics are enhanced (2, 3). However, the harsh wine conditions represent a challenge to the survival and MLF of O. oeni. Therefore, a better understanding of the molecular mechanisms related to the stress tolerance of O. oeni is important for the selection of strains as culture starters (4, 5). Genome sequences of several O. oeni strains are available in the GenBank database, with the O. oeni PSU-1 genome being the only complete sequence reported (6–8). Comparative studies show genetic variation among strains with differences in sugar utilization, amino acid and exopolysaccharide biosynthesis, bacteriophages, and plasmids presence. O. oeni X_2_L (CRL1947; CERELA culture collection, Tucumán, Argentina) was isolated from Malbec red wine of northwest Argentina (9) and selected for its great malolactic activity in the fermentation of Malbec grape must (10). To our knowledge, O. oeni X_2_L is the first published genome of a strain from Argentinian wines.

Total DNA of O. oeni X_2_L was extracted according to the protocol described by Pospiech and Neumann (11). The genome sequence was obtained using a whole-genome shotgun strategy (40-fold genome coverage) with an Ion Torrent personal genome machine (Life Technologies). Quality ﬁltered reads were *in silico* assembled using the DNAstar NGEN assembler, giving 114 large contigs. The draft genome sequence consists of 1,812,711 bp with an average GC content of 37.90%. Genome analysis was performed using the RAST server (12), and tRNA and rRNA genes were annotated by tRNAscan-SE (13) and RNAmmer (14), respectively. Results of RAST analysis showed that there are 247 subsystems represented in the chromosome, which represent only 45% of the sequences assigned. The genome sequence was annotated by the NCBI Prokaryotic Genomes Annotation Pipeline. A total of 1,458 coding DNA sequences, 46 tRNAs, and 6 rRNAs were predicted.

Among genes of biotechnological importance in the wine-making process, the complete cluster of genes related to metabolism of malic acid (*mleA*, *mleP*, *mleR*) and citrate (*citR*, *maeP*, *citC*, *citD*, *citE*, *citF*, *citX*, *citG*) were found in the O. oeni X_2_L genome sequence. Genes encoding for esterases/lipases and glucosidases were also identified. In addition, genes encoding for enzymes involved in the metabolism of nitrogen compounds such as proteases, carboxypeptidases, aminopeptidases, and dipeptidases were detected, whereas genes related to biogenic amines synthesis (histamine, tyramine) were not found, although genes of putrescine transport were identified. The genes predicted through genome analysis showed the genetic potential of O. oeni X_2_L as a starter culture for MLF and its ability to improve sensorial characteristics of red wines.

### Nucleotide sequence accession numbers.

This whole-genome shotgun project has been deposited at DDBJ/EMBL/GenBank under the accession number JROK00000000. The version described in this paper is version JROK01000000.
